# Peritoneal Microbiome in End-Stage Renal Disease Patients and the Impact of Peritoneal Dialysis Therapy

**DOI:** 10.3390/microorganisms8020173

**Published:** 2020-01-25

**Authors:** Liliana Simões-Silva, Ricardo Araujo, Manuel Pestana, Isabel Soares-Silva, Benedita Sampaio-Maia

**Affiliations:** 1 i3S—Instituto de Investigação e Inovação em Saúde, Universidade do Porto, 4200-180 Porto, Portugal; lilianasilva@med.up.pt (L.S.-S.); ricjparaujo@yahoo.com (R.A.); mvasconcelos@hsjoao.min-saude.pt (M.P.); 2 INEB—Instituto de Engenharia Biomédica, Universidade do Porto, Rua Alfredo Allen, 208, 4200-180 Porto, Portugal; 3 Escola Superior de Saúde Dr. Lopes Dias, Instituto Politécnico de Castelo Branco, 6000-767 Castelo Branco, Portugal; 4 Medical Biotechnology, Flinders University of South Australia, Bedford Park SA 5042, Australia; 5 Faculdade de Medicina, Universidade do Porto, 4200-319 Porto, Portugal; 6 Department of Nephrology, Centro Hospitalar Universitário de São João, EPE, 4200-319 Porto, Portugal; 7 Centre of Molecular and Environmental Biology (CBMA), Department of Biology, University of Minho, Campus de Gualtar, 4710-057 Braga, Portugal; 8 Faculdade de Medicina Dentária, Universidade do Porto, 4200-393 Porto, Portugal

**Keywords:** microbiome, peritoneum, chronic kidney disease, peritoneal dialysis, end-stage kidney disease

## Abstract

Factors influencing the occurrence of peritoneal dialysis (PD)-related infections are still far from fully understood. Recent studies described the existence of specific microbiomes in body sites previously considered microbiome-free, unravelling new microbial pathways in the human body. In the present study, we analyzed the peritoneum of end-stage kidney disease (ESKD) patients to determine if they harbored a specific microbiome and if it is altered in patients on PD therapy. We conducted a cross-sectional study where the peritoneal microbiomes from ESKD patients with intact peritoneal cavities (ESKD non-PD, *n* = 11) and ESKD patients undergoing PD therapy (ESKD PD, *n* = 9) were analyzed with a 16S rRNA approach. Peritoneal tissue of ESKD patients contained characteristically low-abundance microbiomes dominated by Proteobacteria, Firmicutes, Actinobacteria, and Bacteroidetes. Patients undergoing PD therapy presented lower species richness, with dominance by the Pseudomonadaceae and Prevotelaceae families. This study provides the first characterization of the peritoneal microbiome in ESKD patients, bringing new insight to the human microbiome. Additionally, PD therapy may induce changes in this unique microbiome. The clinical relevance of these observations should be further explored to uncover the role of the peritoneal microbiome as a key element in the onset or aggravation of infection in ESKD patients, especially those undergoing PD.

## 1. Introduction

The human microbiome influences the well-being of the host by contributing to its nutrition, metabolism, physiology, and immune function, serving to maintain the balance between health and disease states [1,2,3]. Disturbances in the normal gut microbiome are currently associated with the pathogenesis of several chronic diseases, including chronic kidney disease (CKD) [4,5,6,7,8,9,10,11,12]. Gut dysbiosis is well-described in CKD, characterized by lower levels of Bifidobacteriaceae and Lactobacillaceae and higher levels of Enterobacteriaceae [13,14]. The composition of this microbiome is also affected by renal replacement therapy (RRT) used by patients with end-stage kidney disease (ESKD). Dysbiosis in more pronounced in hemodialysis (HD) patients, where increases in potentially pathogenic species and decreases in beneficial species are often observed in patients. This dysbiosis is also present, to a lesser extent, in peritoneal dialysis (PD) patients when compared to controls [14,15].

CKD is an emerging public health problem, with 11–13% of the world’s population afflicted with CKD at any stage (1 to 5) [16,17]. The number of patients with end-stage kidney disease (ESKD) requiring RRT is increasing every year [18]. Major complications of patients undergoing PD include peritonitis and exit-site infections (ESI) [19,20]. However, factors influencing the occurrence of these infections are still far from fully understood.

The most recognized factor contributing to PD patients’ peritonitis is contamination via an exogenous route with skin pathogenic bacteria, such as *Staphylococcus* [21,22,23]. This may occur during connection and disconnection of the dialysis transfer-set. Although microorganisms may access the peritoneum through the catheter exit-site and tunnel, the association between peritonitis and catheter ESI was described in just less than 13% of all peritonitis episodes [24,25,26]. In parallel, endogenous contamination routes of the peritoneal cavity were recognized, including the hematogenous route, vaginal leaking, and translocation of microorganisms through the intestinal epithelial barrier [27,28]. In CKD, impairment of the intestinal epithelial barrier structure and function occurs, thereby facilitating the translocation of intestinal microorganisms, endotoxins, antigens and other microbial products through the intestinal wall toward the systemic circulation and the internal milieu [29,30,31,32,33,34,35]. The blood microbiome was acknowledged and characterized in healthy, non-infectious states [36,37,38,39,40]. Some authors postulated that the hematogenous route was responsible for the colonization of body sites that were previously considered sterile, such as the placenta [41] and breast tissue [42]. Moreover, some authors suggested that the circulating microbiome could be a useful biomarker of cardiovascular risk in CKD patients [43]. Although the peritoneal cavity and surrounding tissues have so far been considered sterile, previous reports described the in vitro intracellular viability of *Staphylococcus aureus* within mesothelial cells, as well as the presence of bacterial DNA and protozoal colonization of peritoneal dialysate in PD patients [44,45,46,47].

In this study, we postulated that the seemingly sterile peritoneum could host its own microbiome. We thus implemented a 16S rRNA next-generation sequencing approach to investigate whether the peritoneum of ESKD patients harbored unique microbiomes that could be altered by PD therapy.

## 2. Materials and Methods

### 2.1. Study Design, Subjects, and Sample Collection

This cross-sectional study included adult ESKD patients who were followed-up in the outpatient clinic of the Nephrology Department of “Centro Hospitalar Universitário de São João” when scheduled for peritoneal catheter insertion, replacement, or removal, or hernia repair. Twenty ESKD adult patients agreed to participate in the study between December 2014 and June 2016, and were distributed into two groups, namely, 11 ESKD patients who were scheduled for peritoneal catheter insertion (ESKD non-PD) and 9 ESKD patients undergoing PD who were scheduled for catheter replacement or removal, hernia repair, or a nephrectomy (ESKD PD). Causes for catheter replacement or removal included peritoneal leakage and catheter failure. Clinical evidence of infection and antibiotic therapy during the preceding month made up the exclusion criteria. The study protocol was approved by the Ethics Committee for Health and Institutional Review Board of “Centro Hospitalar Universitário de São João” (approval number CES-159/11, 2011) and followed the 1964 Helsinki declaration and its later amendments; all recruited patients were asked to give their written informed consent.

Clinical information was gathered from all patients, including age, gender, aetiology of CKD, residual renal function, and time on PD therapy. Information regarding PD-related infectious episodes was gathered in the ESKD PD group before and after peritoneal biopsy collection, and in the ESKD non-PD group after peritoneal tissue collection (i.e., their engagement in PD therapy). Peritoneal tissue samples (1 cm^2^ sections) were collected by trained surgeons under strict sterile conditions in DNA-free sterile microtubes with no visible blood contamination and delivered to the researcher by a nurse. Samples were immediately frozen in liquid nitrogen transport containers and stored at −80 °C.

### 2.2. Specimen Processing and Microbiome Analysis

Genomic DNA was isolated in a strictly controlled environment at Vaiomer (Labège, France); the methodology used was described by Lluch and colleagues [48]. Briefly, peritoneal tissue was mechanical disrupted for 5 s using Ultra-Turrax (IKA, Staufen, Germany), followed by a lysis step using acid-washed glass beads (Sigma, Saint-Louis, MO, USA) and Tissue Lyser (Qiagen, Venlo, The Netherlands) for 2 × 3 min at 30 Hz. After the lysis step, total genomic DNA was extracted using Trizol (Life Technologies, Grand Island, NY, USA). The quality and quantity of DNA extracts were analyzed by agarose gel electrophoresis (1% agarose in TBE 0.5×) and a NanoDrop 2000 UV spectrophotometer (Thermo Scientific, Waltham, MA, USA). PCR amplification was performed using 16S rRNA gene universal primers targeting the V3–V4 region of the bacterial 16S rRNA gene [48]. Illumina sequencing length was designed to encompass the 476 base pair amplicon using the 2 × 300 paired-end MiSeq kit V3. Sample multiplexing was performed using tailor-made 6-bp unique indexes; for each sample, a sequencing library was generated. An equivalent number of raw reads was pooled for each library and the DNA concentration quantified using KAPA Library Quantification Kits for Illumina Platform (Kapa Biosystems, Inc., Wilmington, MA, USA) and 7900HT Fast Real-Time PCR System (Life Technologies). The final pool used for the sequencing showed a concentration between 5 and 20 nM after dilution.

### 2.3. 16S rRNA Gene Sequence Analysis

The targeted microbiome sequences were analyzed using the FROGS bioinformatics pipeline established by Vaiomer (Labège, France) [49]. The following filters were applied: (1) Amplicons with a length <350 nt or a length >480 nt were removed; (2) amplicons without the two PCR primers were removed (10% of mismatches were authorized); (3) amplicons with at least one ambiguous nucleotide (‘N’) were removed; (4) operational taxonomic units (OTU) identified as chimeras (using vsearch v1.9.5 [50]) were removed in all samples; (5) low abundant OTUs (representing ≤0.005% of the whole dataset) were removed; (6) OTUs with strong similarity (≥80%) to phiX were removed. The clustering was performed following two steps with the swarm algorithm v2.1.6 (the first clustering with an aggregation distance of 1 and the second clustering with an aggregation distance of 3). OTU were produced via single-linkage clustering, and taxonomic assignment was performed by Blast+ v2.2.30+ with the databank RDP v11.4. Importing, storage, analysis, and graphical display of the microbiome census data were performed using PhyloSeq v1.14.0. Alpha-diversity was calculated with the (1) observed, (2) Chao1, (3) Shannon, (4) Simpson, and (5) inverse Simpson indices using PhyloSeq v1.14.0. Outputted OTU files were uploaded and formatted for LEfSe [51] analysis using the per-sample normalization of sum values option (http://huttenhower.sph.harvard.edu/galaxy/). The linear discriminant analysis effect size (LEfSe) was conducted using the default values (factorial Kruskal–Wallis test among classes and pairwise Wilcoxon test between subclasses with α = 0.5; the threshold was 2.0 for the logarithmic LDA score) and “all-against-all” for multi-class analysis strategy. LEfSe cladograms were generated, defining Bacteria as the tree root and the differential features detected as biomarkers used to plot abundance histograms. Principal coordinate analysis (PCoA) was performed on the normalized OTU table to compare sample groups/classes based on four methodologies for β-diversity evaluation, namely, (1) Bray–Curtis, (2) Jaccard, (3) Unifrac, and (4) weighted Unifrac.

### 2.4. Statistics

Categorical variables were described through relative frequencies (%) and analyzed using the Chi-square independence test or Fisher’s exact test when more than 1 cell displayed expected counts less than 5. Primer v7 (PRIMER-e, Auckland, New Zealand) was used to calculate the diversity indices, similarity percentages (SIMPER) analysis, and multivariate analysis, mainly ANOSIM and PERMANOVA to test the significance of the beta-diversity. The percentage of OTU data per sample was used for these analyses, followed by square-root transformed data and resemblance matrices of similarity data types using Bray–Curtis similarities, adding dummy values and testing 4999 permutations. Continuous variables were described using mean ± standard deviation (SD) and analysed with Student’s *t* test. *p* < 0.05 was assumed to indicate a significant difference.

### 2.5. Data Availability

The data are available using the link provided by the NCBI team (to access the BioProject PRJNA535341 use the link https://dataview.ncbi.nlm.nih.gov/object/PRJNA535341?reviewer=ptbaa3g7h3e0gj01h3cedk43e6).

## 3. Results

To investigate the existence of microbiomes in the peritoneum of ESKD patients, we generated a cohort of ESKD patients undergoing PD (ESKD PD) and ESKD patients before PD onset (ESKD non-PD). The demographic and clinical characteristics of the two groups of patients included in this study were similar (Table 1).

**Figure 1:**
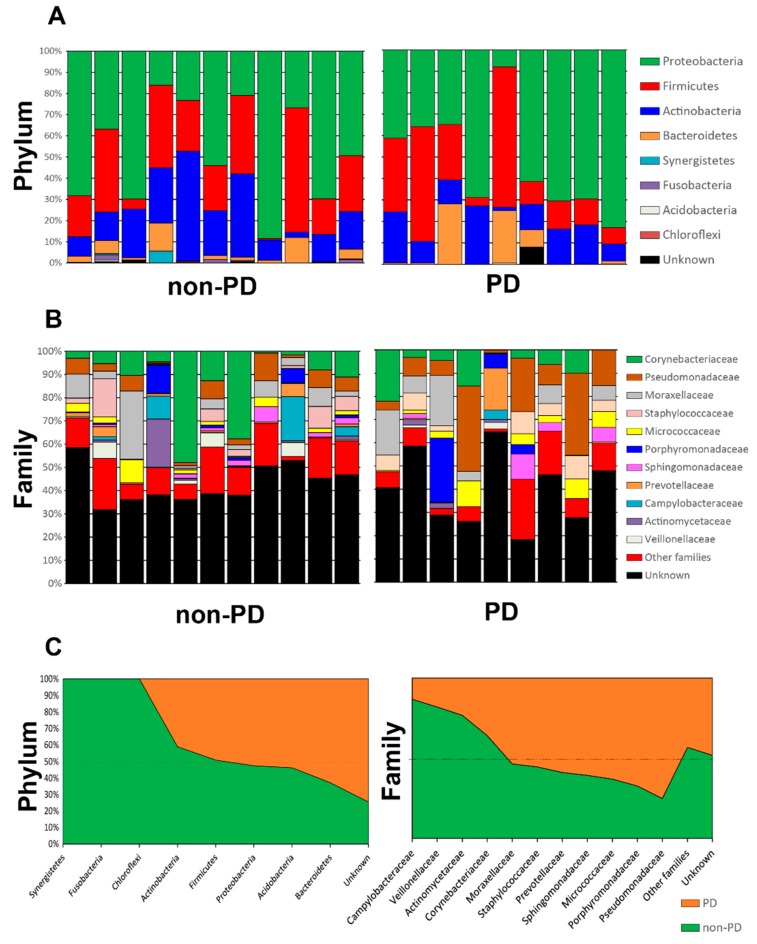
Relative proportion taxa for taxonomic-level phylum (**A**) and family (**B**); the averages for the groups of patients (non-PD versus PD) are shown in (**C**).

Both groups were subjected to bacterial microbiome analyses of peritoneal tissue. Samples displayed a median of 39,116 reads (range: 33,085–46,171), of which 64.2%± 2.7% were classified into OTUs or amplicon single variants (ASVs). A total of 329 OTUs were detected (median of 62 OTUs per sample) with samples presenting between 24 and 129 OTUs; a total of 423 ASVs were found (with a median of 50). The rarefaction curve analysis demonstrated the sufficiency of our read coverage to capture sample diversity (Figure S1). OTUs were clustered with the Swarm algorithm, revealing the relative proportion of different taxonomic levels in each individual sample (Figure 1 and Figure S2). The taxonomic profiles of the peritoneal tissue microbiome in ESKD non-PD and ESKD PD were similar at high taxonomic levels, dominated by Proteobacteria, Firmicutes, Actinobacteria, and Bacteroidetes (Figure 1). Taxonomic differentiation of these two groups of patients was nicely resolved at the family level and below, with an overrepresentation of Corynebacteriaceae and Campylobacteriaceae in ESKD non-PD patients (Figure 1).

**Figure 2:**
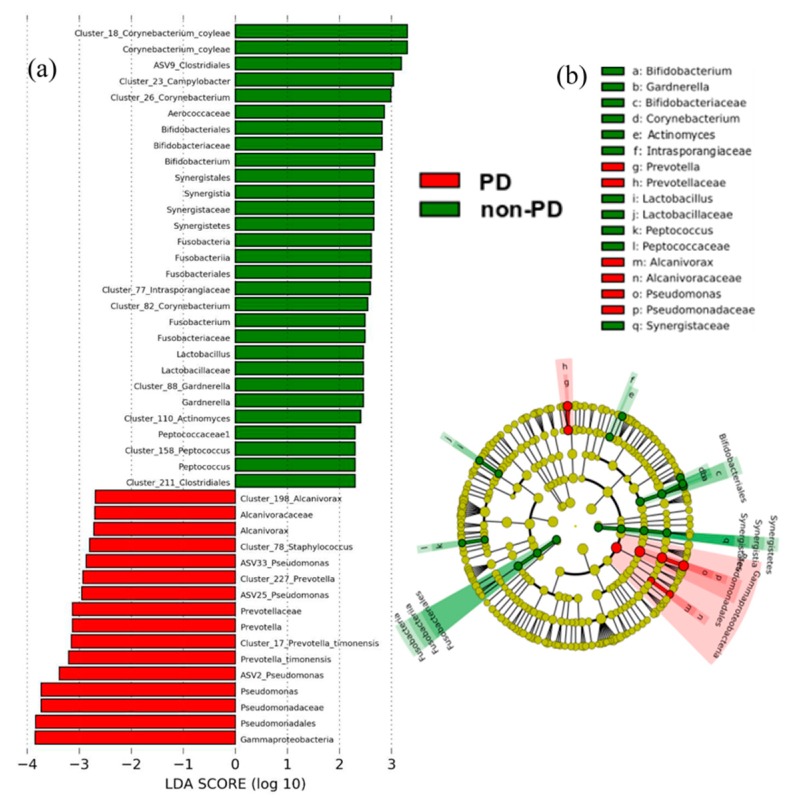
Microbiome-derived bacterial taxa and OTUs identified as differentially abundant between ESKD PD or non-PD patients; data were analyzed by linear discriminate analysis coupled with effect size measurements (LEfSe) (**a**) and projected as a cladogram (**b**). Taxa with a nominal *p* value of <0.05 are highlighted on the cladogram in red and green, indicating significant differences between the groups of non-PD and PD patients; operational taxonomic units (OTUs) are mentioned as clusters. ASV: amplicon single variant.

In agreement, the microbiome biomarker discovery approach called linear discriminate analysis coupled with effect size measurements (LEfSe) revealed that ESKD non-PD patients showed an enrichment of the microbial families Corynebacteriaceae, Bifidobacteriaceae, Lactobacillaceae, Synergistaceae and Peptococcaceae compared to ESKD PD patients (see statistics and cladogram in Figure 2; details shown in barplot abundances in Figure S3). In ESKD PD, the lack of the previous bacteria resulted in the promotion of Pseudomonadaceae, Prevotellaceae, and Alcanivoracaceae, specifically from the genera *Pseudomonas*, *Prevotella,* and *Alcanivorax*.

Similar results were observed with similarity percentages (SIMPER) analysis, reinforcing the characterization of ESKD PD samples by multiple OTUs of *Prevotella*, *Pseudomonas,* and *Staphylococcus*, while samples from ESKD non-PD patients were mostly characterized by OTUs classified as *Corynebacterium*, *Bifidobacterium*, *Gardnerella,* and *Acinetobacter*.

**Figure 3:**
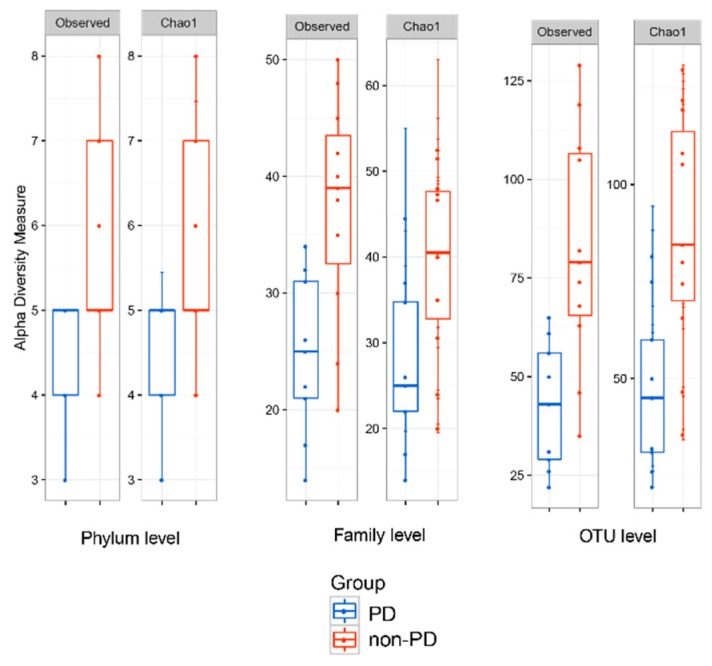
Alpha-diversity of the peritoneal microbiome community at the phylum, family, and OTU taxonomic levels using the observed (left panels) and Chao1 (right panels) indices.

Alpha-diversity analysis was estimated across different indexes (Figure 3 and Figure S4) and presented statistical significance using the observed and Chao1 analyses; the Shannon diversity indices for the complete microbiome in ESKD non-PD and PD patients were 3.83 and 3.20, respectively. Collectively, alpha-diversity analysis revealed that ESKD non-PD patients presented higher bacterial species richness than ESKD PD patients, with the values for Margalef richness calculated at 46.1 and 27.1, respectively.

**Figure 4:**
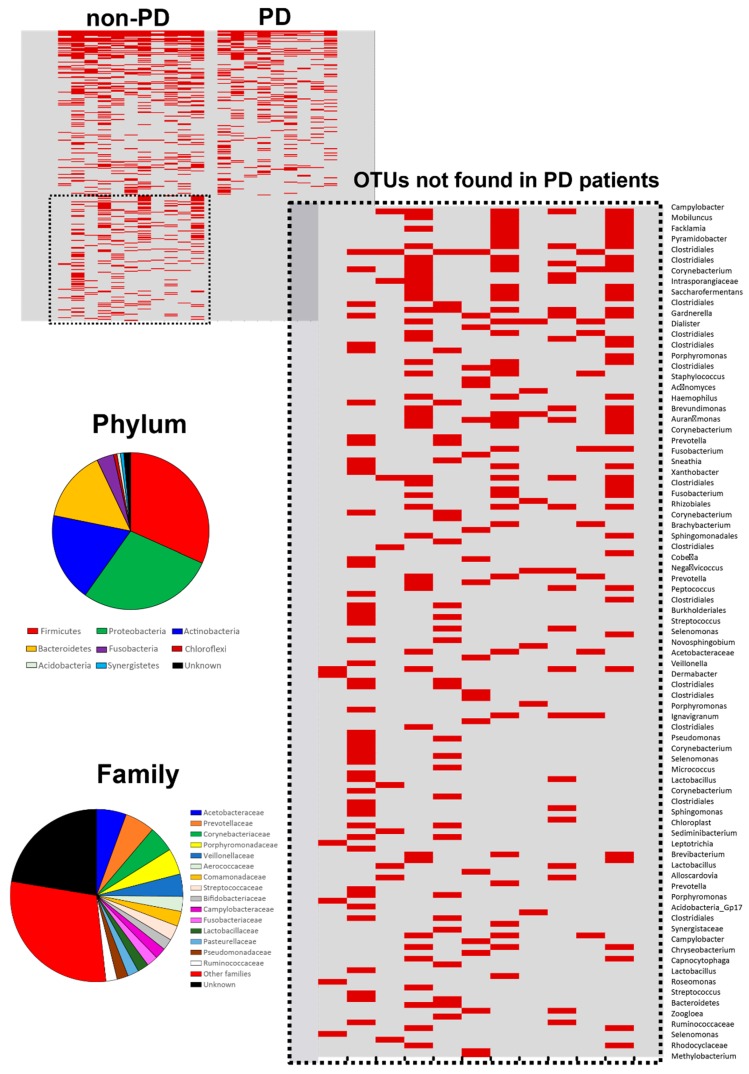
Heat map showing OTU presence (in red) and absence in all the peritoneum samples from ESKD PD and ESKD non-PD (column: sample; row: OTUs). Details of the microbiome lacking in PD patients, including the identification of each OTU and its classification at the phylum and family levels.

The taxa lost in the PD patients were mostly identified as belonging to the major groups of Firmicutes, Proteobacteria, Actinobacteria, and Bacteroidetes, and to the families Acetobacteraceae, Prevotellaceae, Corynebacteriaceae, Porphyromonadaceae, and Veillonellaceae (Figure 4). An additional 40 families were also identified in this missing microbial population; the OTUs absent in the ESKD PD patients consisted mainly of rare OTUs not present in all non-PD patients (Figure 4).

**Figure 5:**
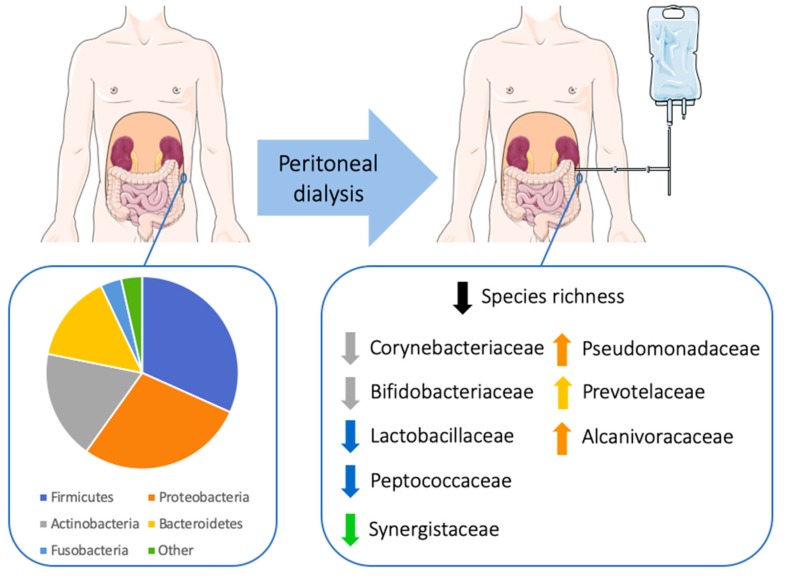
Schematic representation of the peritoneal microbiome at the phylum level in end-stage kidney disease (ESKD) patients, and the impact of peritoneal dialysis on the peritoneal microbiome. Figure was produced using Servier Medical Art (http://smart.servier.com/).

Overall, ESKD PD patients presented lower peritoneal microbiome richness, lacking many OTUs belonging to the families Corynebacteriaceae, Bifidobacteriaceae, Lactobacillaceae, Synergistaceae, and Peptococcaceae found in non-PD patients and resulting in the prominence of other OTUs belonging mainly to the Pseudomonadaceae, Prevotellaceae, and Alcanivoracaceae families (Figure 5).

Principal coordinate analysis (PCoA) for the beta-diversity assessment did not show differences in the microbial communities in the peritoneal tissues between ESKD PD and ESKD non-PD groups (Figure S5), indicating that the observed differences were mainly associated with rare and/or less abundant lineages. Analysis of similarities (ANOSIM) and permutational multivariate analysis of variance (PERMANOVA) confirmed the PCoA observations, as the values of both analyses were not significant (*p* = 0.1).

**Figure 6:**
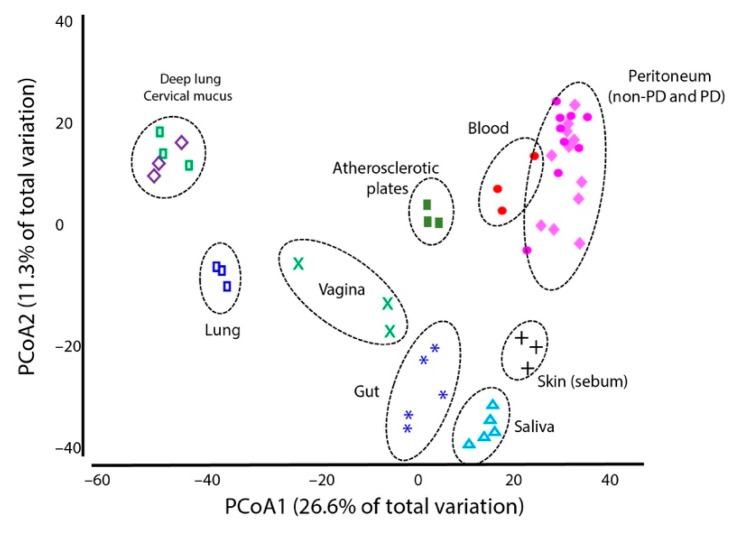
Principal coordinates analysis (PCoA) of multiple human microbiomes in addition to the peritoneal microbiomes of non-PD (pink diamonds) and PD (pink circles) patients described in this study. The graph was produced in Primer 7 software using the microbiome information for the genera (total reads per taxa were converted to a percentage for each sample), square-root transformed data, and resemblance matrices (similarity data types using Bray–Curtis similarities and adding dummy values). The information for the multiple microbiomes was obtained from the European Bioinformatics Institute (EMBL-EBI) public databases; random samples were selected from the following microbiome projects: MGYS00001556, MGYS00002219, MGYS00001070, MGYS00002184, ERS1066787, MGYS00002073, MGYS00001168, and MGYS00001695.

When comparing the peritoneal microbiome and microbiomes in other parts of the human body, differences were observed; the blood microbiome presented the closest profile to the peritoneal microbiome (Figure 6 and Figure S6). However, analysis of similarities (ANOSIM) and permutational multivariate analysis of variance (PERMANOVA) confirmed that the peritoneal and blood microbiomes were significantly distinct (*p* < 0.01).

Regarding the infectious episodes of ESKD patients from the study group, most of the observed PD-related infectious episodes were ESIs, with *Corynebacterium* and *Staphylococcus* spp. being the main infectious agents (Table 2).

These infectious agents represented genera and species commonly found in the peritoneal microbiomes of ESKD non-PD and PD patients. Moreover, all of the observed infectious agents were within the 10 most prevalent genera comprising the peritoneal microbiome of each patient, except for the genus *Streptococcus*, which was included in the rare fraction of the peritoneal microbiome.

Two patients (each categorized as either ESKD non-PD or ESKD PD) showed atypical high frequency values for *Corynebacterium* (38% and 10%, respectively) in the microbiome biopsy, with specific OTUs contributing to the values; these OTUs were rarely found in other patients and, curiously, in both cases, the patients displayed *Corynebacterium* infections in the following month. Another patient with an atypically high frequency of *Staphylococcus* (17%) in the microbiome biopsy, once again mostly attributed to a single OTU, was reported to suffer a *Staphylococcus* infection three months later. In all cases, we did not have access to the infectious agent isolate later in order to confirm its presence in the microbiome when the peritoneal sample was collected.

## 4. Discussion

To the best of our knowledge, this is the first study to report the existence of a microbiome in the peritoneum of ESKD patients. The peritoneal microbiome was dominated by Proteobacteria and Firmicutes, followed by Actinobacteria and Bacteroidetes. In ESKD patients submitted to PD, the peritoneal microbiome presented lower richness and an increased dominance of the Pseudomonadaceae and Prevotellaceae families.

A previous study reporting a microbiome dominated by Proteobacteria, Actinobacteria, Firmicutes, and Bacteroidetes in peritoneal tumors of *Pseudomyxoma peritonei* corroborated our results [52]. In line with our findings, previous studies discovered microbiomes in body sites that were previously considered microbiome-free, such as the placenta [41] and breast tissue [42]. Collectively, these studies suggested a scenario of mobility pathways of the human microbiome through different body habitats. This atopobiosis (microbes that appear in places other than their normal locations) has long been recognized as an important route for endogenous infections and, more recently, was implicated in the pathogenesis of a variety of inflammatory diseases [36].The fact that peritoneal tissue of ESKD patients harbors unique, low-abundance microbiomes dominated by Proteobacteria and Firmicutes may be linked to the increased microbial intestinal translocation observed in these patients [29,30,31]. All of these phyla were found in the intestinal microbiota of ESKD patients [13,53]. Recently, the blood microbiome was also described in ESKD patients to be dominated by Proteobacteria, followed by Firmicutes and Bacteroidetes [54]. There are some similarities and differences between these microbiomes, with the abundance of Proteobacteria, especially *Pseudomonas*, one of the largest similarities [40]. The peritoneal microbiome could be comparable to some extent to the blood microbiome in contrast to other microbiomes described in other human body locations, suggesting that the blood microbiome may represent a source for the peritoneal microbiome. The peritoneal microbiome described for ESKD patients had some similarities to the blood microbiome of unrelated individuals. A possible contamination from the patients’ blood during peritoneal biopsy cannot be excluded, although all precautions were taken to avoid it. Nevertheless, the peritoneal and blood microbiomes presented distinct features, as shown by the ANOSIM and PERMANOVA statistical tests.

In ESKD, PD therapy could represent a new pathway, per se, for microorganisms to invade the human body, either through close contact with the skin microbiome due to the existence of a catheter connecting the skin and the peritoneal cavity, or via the presence of the PD catheter biofilm. However, we did not observe evidence of similarities between the peritoneal and skin microbiomes, since the most abundant colonizer organisms of umbilical and inguinal crease, *Staphylococcus* and *Corynebacterium* spp. [1,55], were not significantly altered or increased in ESKD PD patients in comparison to ESKD non-PD patients. The existence of biofilms on PD catheters could explain the increase in Pseudomonaceae in the peritoneal microbiomes of ESKD PD patients [56,57]. However, this explanation is probably incorrect, as no new OTUs of *Pseudomonas* were generally found in ESKD PD patients; the variation observed in PD patients is an increase in OTUs also present in non-PD patients. The increased amount of *Pseudomonas* observed in PD patients most likely represented a consequence of lower bacterial richness and lack of other OTUs to balance out the microbiomes in these patients, which was a feature described for non-PD patients. Regardless, other factors may alter the peritoneum environment, favoring certain species to the detriment of others, including dialysis solution composition, frequent daily exchanges, and pressure variations.

In PD patients, it is especially critical to understand the role of the peritoneal microbiome in the development of PD-related infections due to patients’ local immune impairments [58]. The majority of the infectious agents found in these patients were within the 10 most prevalent genera of the newly described peritoneal microbiome. In a few cases, it was possible to identify abundant OTUs in the patients’ biopsies few months before the patient showed any symptoms of infection; this observation was done for *Corynebacterium* and *Staphylococcus*. Altogether, this may suggest a possible link between peritoneal microbiome and posterior PD-related infections, but further genotyping or metagenomic approaches should be performed before this link can be established. The potential of this methodology in clinical diagnostics and infection prevention was suggested by other researchers and clinicians [59].

The dominance of Pseudomonadaceae in the peritoneal microbiome of PD patients could be of major clinical importance, given that patients with Gram-negative peritonitis have worse clinical outcomes [60], are more likely to require hospital admission, and can experience catheter loss and failure of PD [60,61,62,63,64,65]. *Pseudomonas* spp. are probably the most relevant agents of severe peritonitis in ESKD PD patients [60]. However, within our patient population, we did not observe Pseudomonadaceae infections either before or after biopsy sampling. Some Actinobacteria, in particular *Bifidobacterium* and *Actinomyces*, are known to competitively protect against pathogenic bacteria by preventing adherence to enterocytes in the gut and competing for nutrients, iron, and other essential ions [66]. This “downregulation” of *Corynebacterium*, *Bifidobacterium*, *Gardnerella*, and other bacteria on the peritoneal microbiome of ESKD PD patients is a noteworthy result that deserves further investigation. Such studies could also be extended to the peritoneal dialysate microbiome profile, as we previously reported the asymptomatic colonization of protozoa in the peritoneal dialysate of PD patients [47], proposing that some organisms not necessarily linked to infectious states may have been present in these samples.

We acknowledge that there were some gender differences between the non-PD and PD groups and we cannot exclude the existence of gender differences in the peritoneal microbiome. Nevertheless, gender differences were previously described for some Bacteroidetes (*Bacteroides*, *Prevotella*) and Firmicutes (*Turicibacter*), namely that these groups were more prevalent in the gut microbiomes of males and females, respectively [67,68,69]. None of these taxa played a large role in PD patients, as most of the differences were found in other taxa.

Although this study included a limited number of samples, it represents a starting point to uncover the role of the peritoneal microbiome in health and disease. Furthermore, gathering information on the peritoneal microbiome of healthy, non-ESKD patients would certainly be not only interesting, but helpful to better understand the impact of this microbiome on health and, consequently, on CKD. Therefore, further studies evaluating the peritoneal microbiome in a health context are required.

## 5. Conclusions

The peritoneum of ESKD patients harboured specific microbiomes; PD therapy may be associated with changes in the microbiome, namely, an increased dominance of the Pseudomonadaceae and Prevotellaceae families. Overall, this study brings new insight to the characterization of the human microbiome. Considering that the peritoneum has its own microbiome, there is still a long way to go before we fully understand its role in health and disease. The peritoneal microbiome in particular can contribute to the onset or aggravation of infections in ESKD patients and inflammation in PD patients. Human microbiome modulation, particularly in regard to the peritoneal microbiome, may represent a future therapeutic target in this patient population.

## Figures and Tables

**Table 1:** Age, gender, aetiology of CKD and time on peritoneal dialysis of ESKD patients undergoing PD (ESKD PD) and ESKD patients (ESKD non-PD).

	ESKD PD (*n* = 9)	ESKD Non-PD (*n* = 11)	*p* Value
Age (years)	45.11 ± 11.27	43.27 ± 12.42	0.736
Gender (male, %):			0.070
Male (%)	77.8%	23.7%	
Female (%)	22.2%	72.7%	
Aetiology of CKD (%):			0.515
Glomerular disease (GD)	44.4%	45.5%	
Diabetic nephropathy	11.1%	18.2%	
Other GD	33.3%	27.3%	
Tubulointerstitial disease	22.2%	45.5%	
ADPKD	22.2%	18.2%	
Other TID	0%	27.3%	
Vascular disease	11.1%	9.1%	
Unknown aetiology	22.2%	0%	
Time on PD (months)	20.37 ± 24.97	-	

Results are shown in prevalence (%) or mean ± standard deviation (SD). ESKD: end-stage renal disease; CKD: chronic kidney disease; PD: peritoneal dialysis; GD: glomerular disease; ADPKD, Autosomal Dominant Polycystic Kidney Disease; TID, tubulointerstitial disease.

**Table 2:** Bacterial infection history of ESKD patients (ESKD PD and ESKD non-PD), namely peritonitis and exit-site infections 12 months before and 12 months after peritoneal biopsies (samples used for the microbiome study).

Infection History	ESKD PD (*n* = 9)		ESKD Non-PD (*n* = 11)
	Before Biopsy	After Biopsy	After Biopsy
Peritonitis	*Streptococcus mitis*/*oralis* (1)		*Staphylococcus aureus* (1) *S. mitis*/*oralis* (1) Not identified (2)
Exit-site infections	*Corynebacterium* (2) *Corynebacterium xerosis* (1) Not identified (2)	*Corynebacterium* (1) *Corynebacterium simulans* (1) Not identified (1)	*Corynebacterium* (2) *S. aureus* (1) Not identified (4)

Results are expressed as “agent (number of patients)”. ESKD: end-stage kidney disease.

## Supplementary Materials

The following are available online at https://www.mdpi.com/2076-2607/8/2/173/s1, Figure S1 (Rarefaction curves), Figure S2 (Relative proportion taxa for class, order, genus, and species), Figure S3 (Abundances of the genera and families between the ESRD-PD and ESRD-nonPD), Figure S4 (Alpha diversity of the peritoneum microbiome community at phylum, class, order, family, genus, species and OUT taxonomic levels calculated with Observed, Chao1, Shannon, Simpson, and Inverse Simpson indexes), Figure S5 (Beta-diversity of the peritoneum microbiome community at OTU level with Bray-Curtis, Jaccard, unweighted Unifrac, and Weighted Unifrac) and Figure S6 (Non-metric multidimensional scaling of blood and peritoneum microbiomes of non-PD and PD patients).
