# Supplementary material for: Peritoneal Microbiome in End-Stage Renal Disease Patients and the Impact of Peritoneal Dialysis Therapy
Source: Microorganisms. 2020 Jan 25;8(2):173. doi: 10.3390/microorganisms8020173 (PMC7074711; doi:10.3390/microorganisms8020173)
Supplement: Supplementary file 1 [file microorganisms-08-00173-s001.zip › microorganisms-650202-supplementary.pdf]

Supplementary material

Peritoneal Microbiome in End-Stage Renal Disease Patients and the Impact of Peritoneal Dialysis Therapy

Liliana Simões-Silva, Ricardo Araujo, Manuel Pestana, Isabel Soares-Silva, Benedita Sampaio-Maia

Table of contents **Pag.**

Figure S1: Rarefaction curves…………………………...…………………………………………… 2

Figure S2: Relative proportion taxa for class, order, genus, and species…………………………... 3

Figure S3: Abundances of the genera and families between the ESRD-PD and ESRD-nonPD….. 4

Figure S4: Alpha diversity of the peritoneum microbiome community at phylum, class, order, family, genus, species and OUT taxonomic levels calculated with Observed, Chao1, Shannon, Simpson, and Inverse Simpson indexes………………………………………………………………….. 16

Figure S5: Beta-diversity of the peritoneum microbiome community at OTU level with Bray-Curtis, Jaccard, unweighted Unifrac, and Weighted Unifrac ………………………………………….. 18

Figure S6: Non-metric multidimensional scaling of blood and peritoneum microbiomes of non-PD and PD patients ……………..………………………………………………………………….. 19

Figure S1

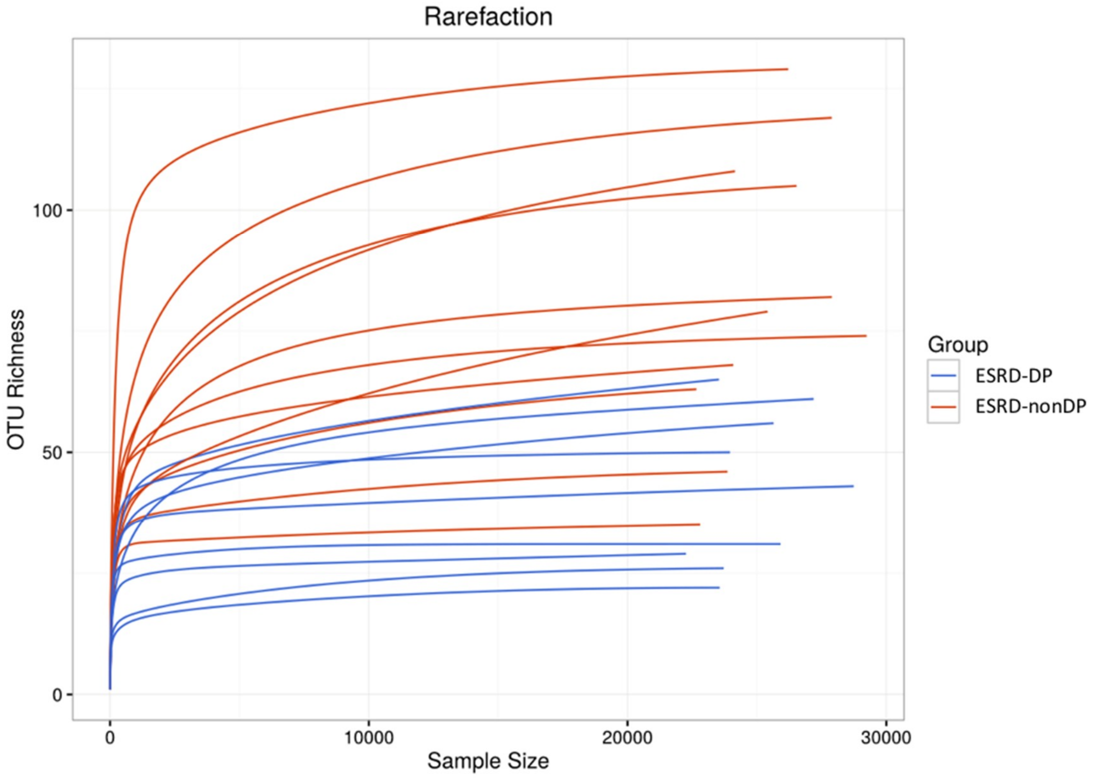

Figure S1: The rarefaction curves plotted with all groups cumulatively. The rarefaction analysis curves suggest the sample diversity was captured as expected given the average sequence pairs classified into operational taxonomic units (OTU).

Figure S2

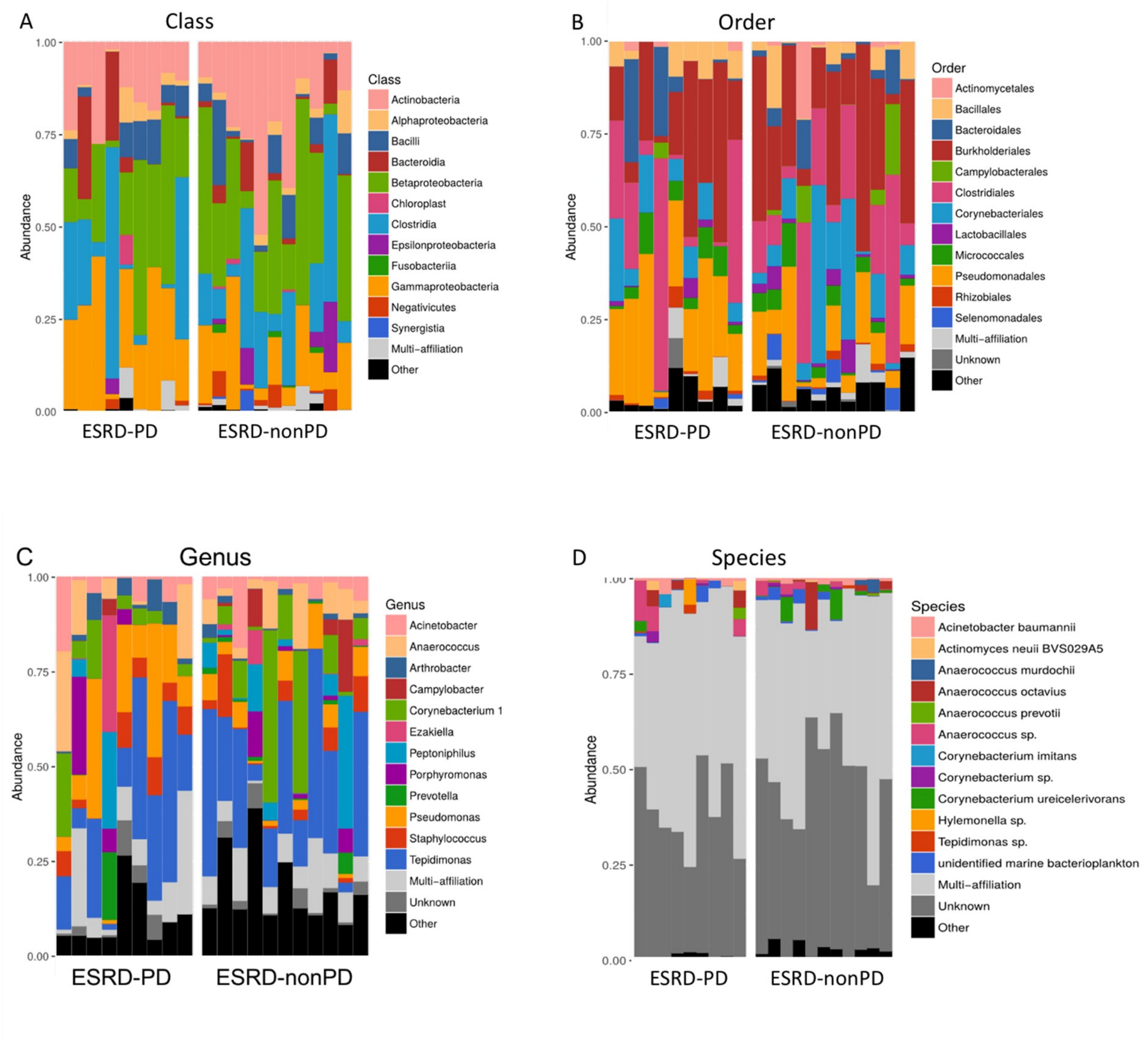

Figure S2: Relative proportion taxa for class (A), order (B), genus (C), and species (D). Taxa are identified by name in the plot for abundance >1%. Taxa are merged into the "Other" category only if it exists in any sample with abundance greater than 0.01%. Taxa are merged into the "Multi-affiliation" category when they can correspond to two or more different taxa. ESRD-PD – End-stage renal disease patients undergoing peritoneal dialysis; ESRD-nonPD – End-stage renal disease patients with intact peritoneal cavity.

Figure S3

A Bacteria.Actinobacteria.Actinobacteria.Actinomycetales.Actinomycetaceae.Actinomyces

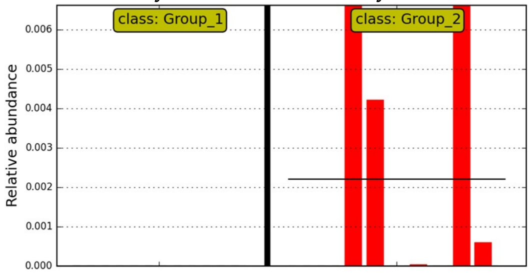

B Bacteria.Actinobacteria.Actinobacteria.Actinomycetales.Actinomycetaceae.Actinomyces.Unknown.Cluster_110

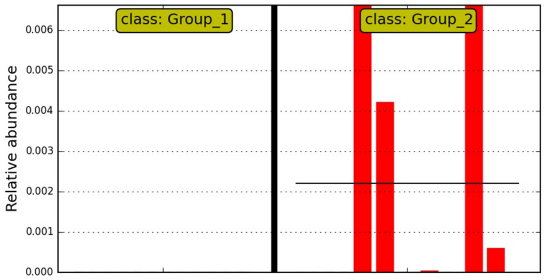

C Bacteria.Actinobacteria.Actinobacteria.Bifidobacteriales

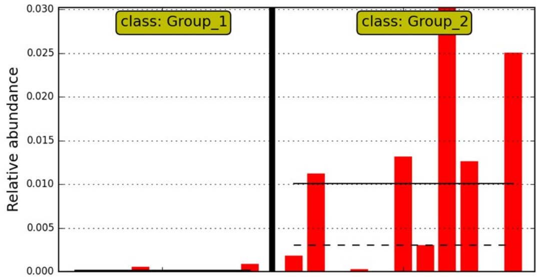

D Bacteria.Actinobacteria.Actinobacteria.Bifidobacteriales.Bifidobacteriaceae

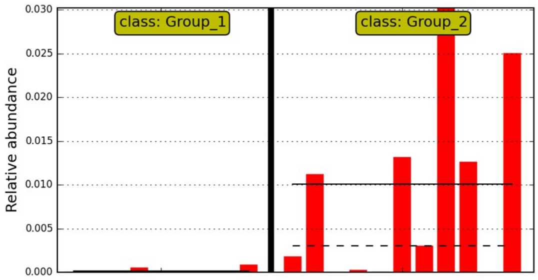

E Bacteria.Actinobacteria.Actinobacteria.Bifidobacteriales.Bifidobacteriaceae.Bifidobacterium

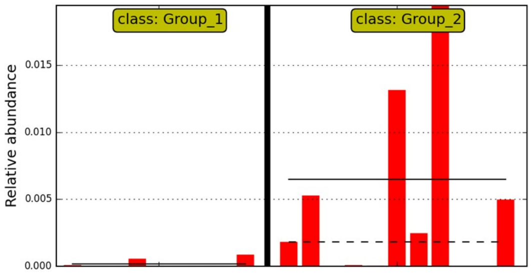

F Bacteria.Actinobacteria.Actinobacteria.Bifidobacteriales.Bifidobacteriaceae.Bifidobacterium.Multi_affiliation

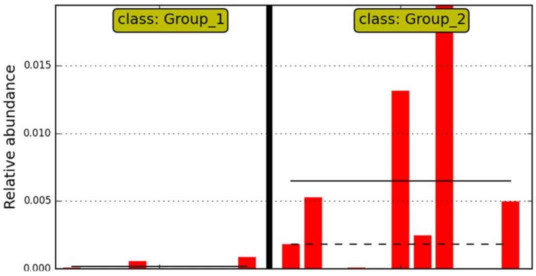

G Bacteria.Actinobacteria.Actinobacteria.Bifidobacteriales.Bifidobacteriaceae.Gardnerella

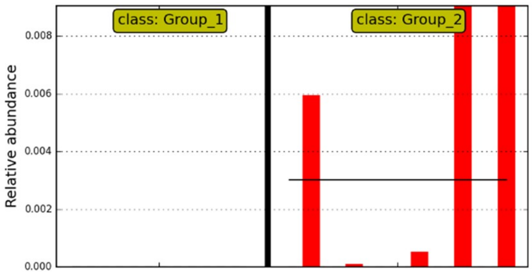

H Bacteria.Actinobacteria.Actinobacteria.Bifidobacteriales.Bifidobacteriaceae.Gardnerella.Multi_affiliation

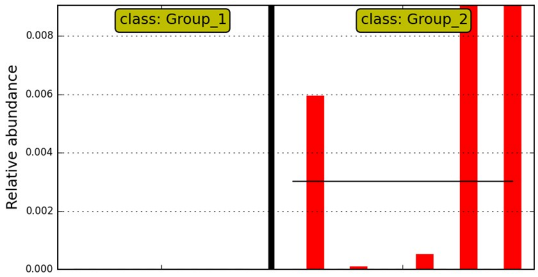

I Bacteria.Actinobacteria.Actinobacteria.Bifidobacteriales.Bifidobacteriaceae.Gardnerella.Multi_affiliation.Cluster_88

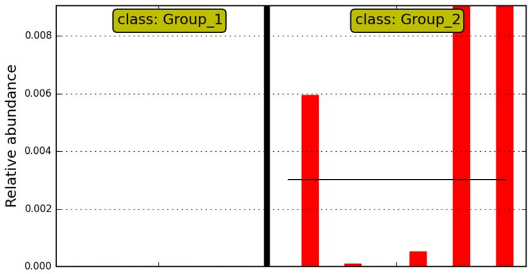

J Bacteria.Actinobacteria.Actinobacteria.Corynebacteriales.Corynebacteriaceae.Corynebacterium

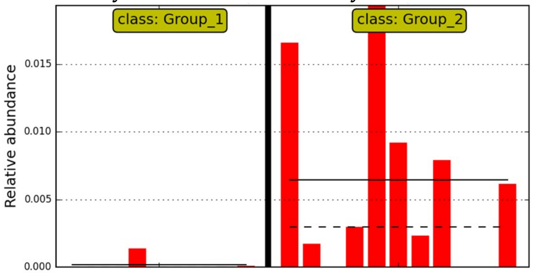

K Bacteria.Actinobacteria.Actinobacteria.Corynebacteriales.Corynebacteriaceae.Corynebacterium1.Corynebacteriumureicelerivorans

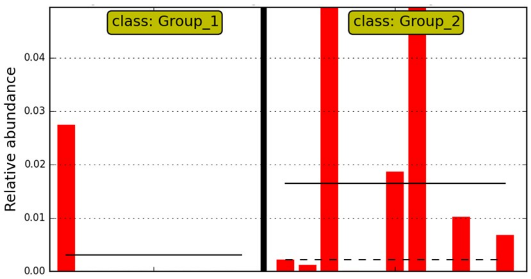

L Bacteria.Actinobacteria.Actinobacteria.Corynebacteriales.Corynebacteriaceae.Corynebacterium1.Corynebacteriumureicelerivorans.Cluster_26

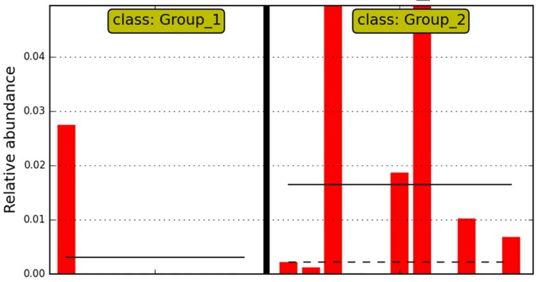

M Bacteria.Actinobacteria.Actinobacteria.Corynebacteriales.Corynebacteriaceae.Corynebacterium1.Unknown.Cluster_18

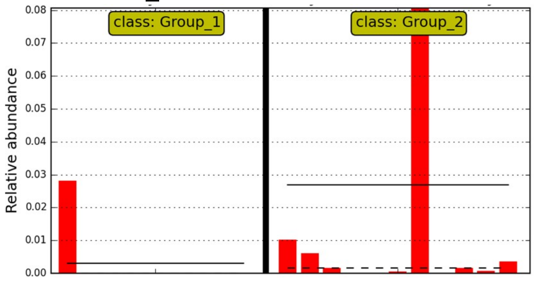

N Bacteria.Actinobacteria.Actinobacteria.Corynebacteriales.Corynebacteriaceae.Corynebacterium.Multi_affiliation

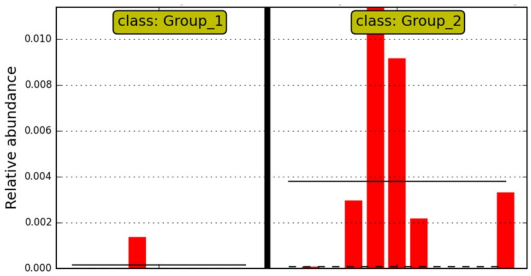

O Bacteria.Actinobacteria.Actinobacteria.Corynebacteriales.Corynebacteriaceae.Corynebacterium.Multi_affiliation.Cluster_82

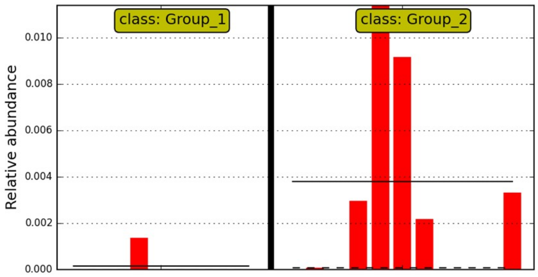

P Bacteria.Actinobacteria.Actinobacteria.Micrococcales.Multi_affiliation

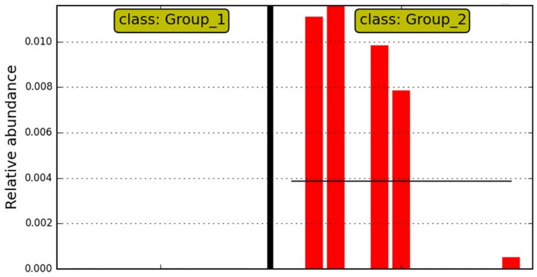

Q Bacteria.Actinobacteria.Actinobacteria.Micrococcales.Multi_affiliation.Multi_affiliation

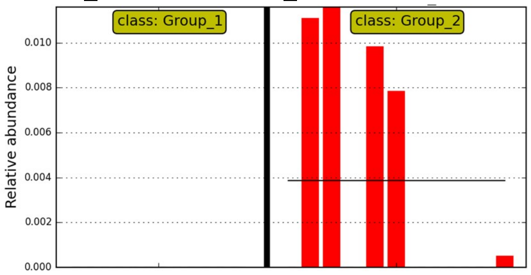

R Bacteria.Actinobacteria.Actinobacteria.Micrococcales.Multi_affiliation.Multi_affiliation.Multi_affiliation

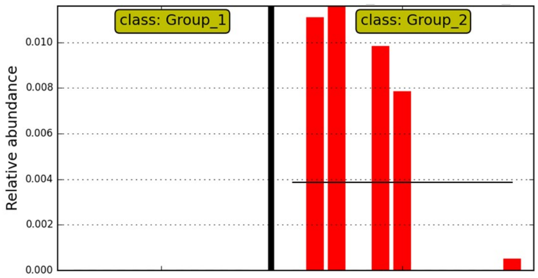

S Bacteria.Actinobacteria.Actinobacteria.Micrococcales.Multi_affiliation.Multi_affiliation.Multi_affiliation.Cluster_77

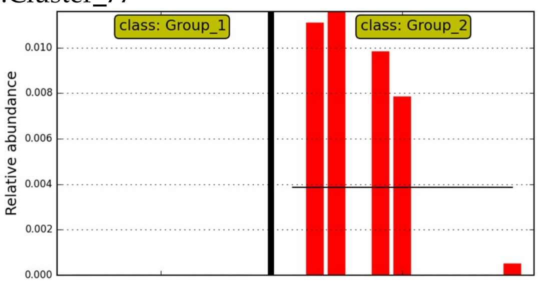

T Bacteria.Bacteroidetes.Bacteroidia.Bacteroidales.Prevotellaceae

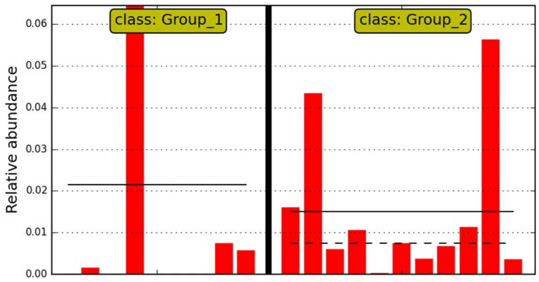

U Bacteria.Bacteroidetes.Bacteroidia.Bacteroidales.Prevotellaceae.Prevotella

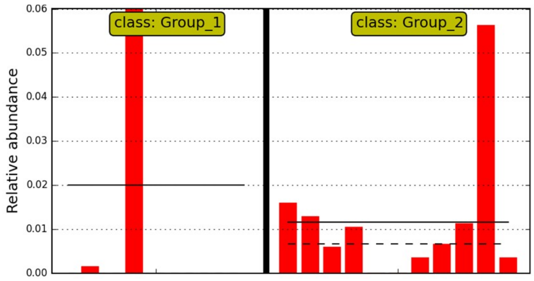

V Bacteria.Bacteroidetes.Bacteroidia.Bacteroidales.Prevotellaceae.Prevotella7.Prevotellasp_

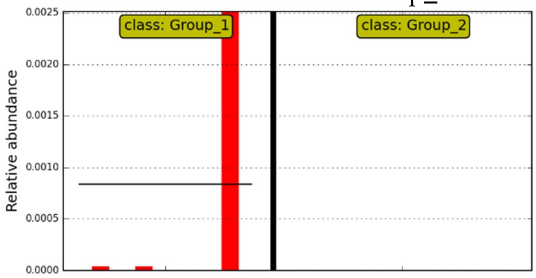

W Bacteria.Bacteroidetes.Bacteroidia.Bacteroidales.Prevotellaceae.Prevotella7.Prevotellasp_.Cluster_227

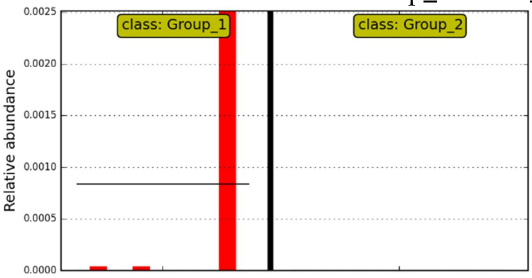

X Bacteria.Bacteroidetes.Bacteroidia.Bacteroidales.Prevotellaceae.Prevotella.Multi_affiliation

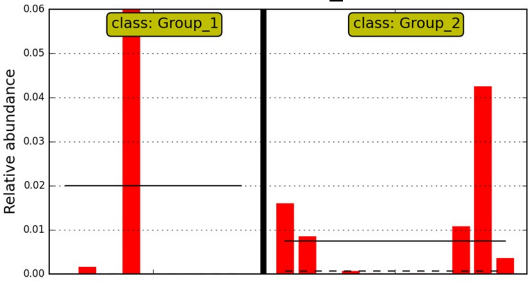

Y Bacteria.Bacteroidetes.Bacteroidia.Bacteroidales.Prevotellaceae.Prevotella.Multi_affiliation.Cluster_17

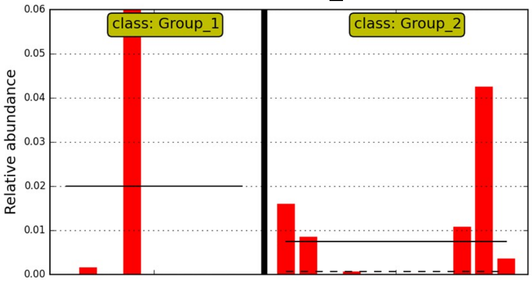

Z Bacteria.Bacteroidetes.Bacteroidia.Bacteroidales.Prevotellaceae.Prevotella.Unknown

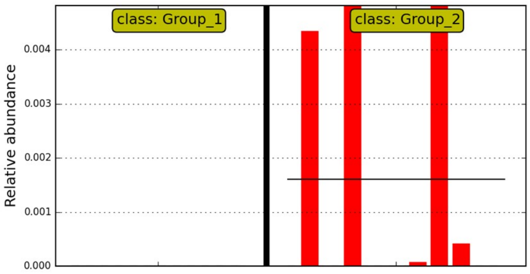

AA Bacteria.Firmicutes.Bacilli.Bacillales.Staphylococcaceae.Staphylococcus.Multi_affiliation.Cluster_78

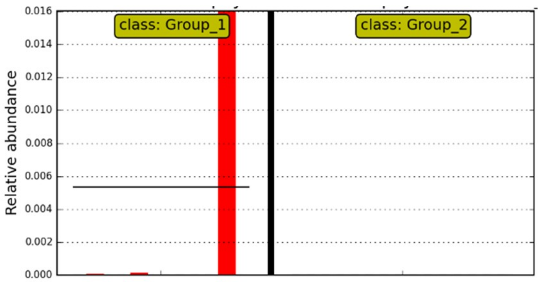

AB Bacteria.Firmicutes.Bacilli.Lactobacillales.Lactobacillaceae

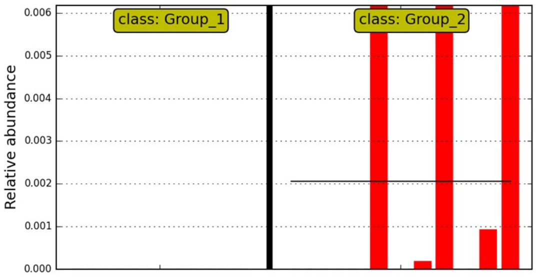

AC Bacteria.Firmicutes.Bacilli.Lactobacillales.Lactobacillaceae.Lactobacillus

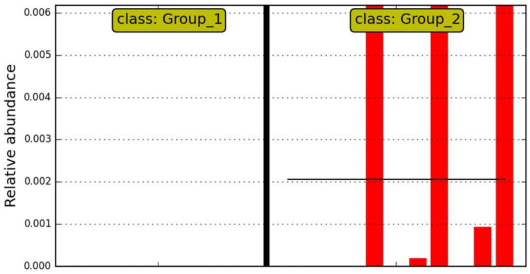

AD Bacteria.Firmicutes.Bacilli.Lactobacillales.Lactobacillaceae.Lactobacillus.Multi_affiliation

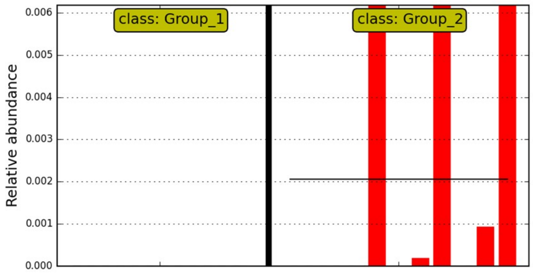

AE Bacteria.Firmicutes.Clostridia.Clostridiales.FamilyXI.Peptoniphilus.CandidatusPeptoniphilusmassiliensis

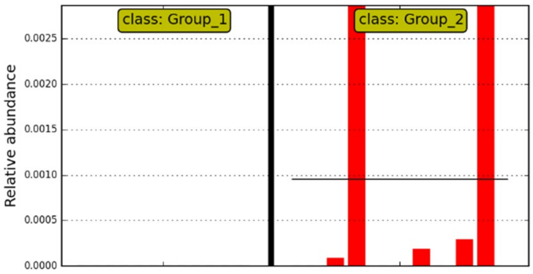

AF Bacteria.Firmicutes.Clostridia.Clostridiales.FamilyXI.Peptoniphilus.CandidatusPeptoniphilusmassiliensis.Cluster_211

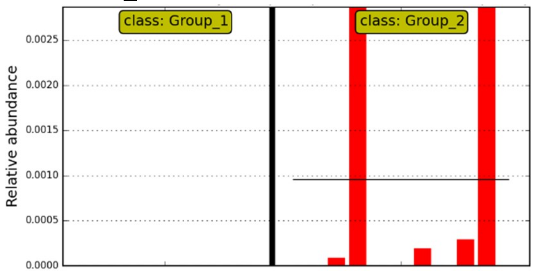

AG Bacteria.Firmicutes.Clostridia.Clostridiales.Peptococcaceae

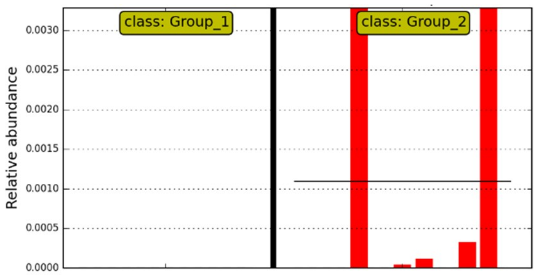

AH Bacteria.Firmicutes.Clostridia.Clostridiales.Peptococcaceae.Peptococcus

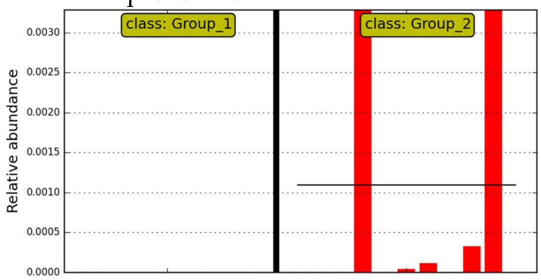

AI Bacteria.Firmicutes.Clostridia.Clostridiales.Peptococcaceae.Peptococcus.Unknown

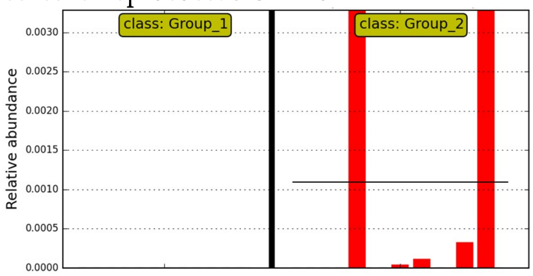

AJ Bacteria.Firmicutes.Clostridia.Clostridiales.Peptococcaceae.Peptococcus.Unknown.Cluster_158

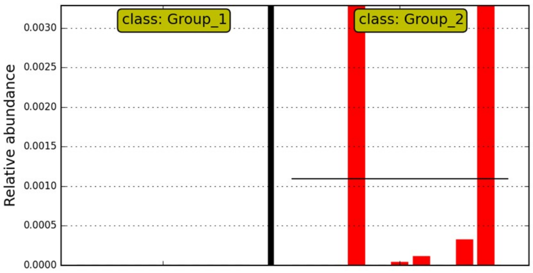

AK Bacteria.Fusobacteria

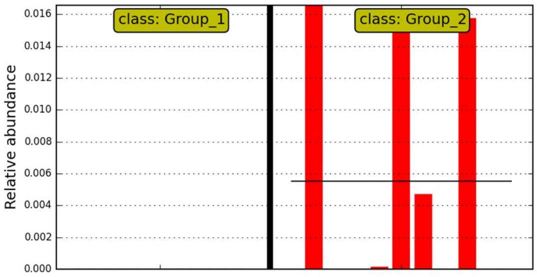

Bacteria.Fusobacteria.Fusobacteriia

AL

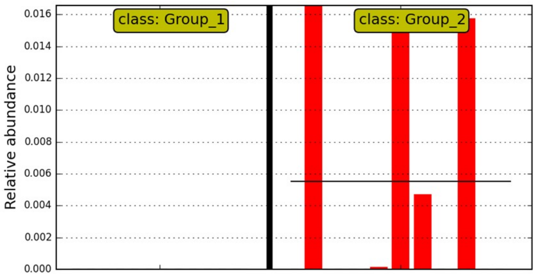

AM Bacteria.Fusobacteria.Fusobacteriia.Fusobacteriales

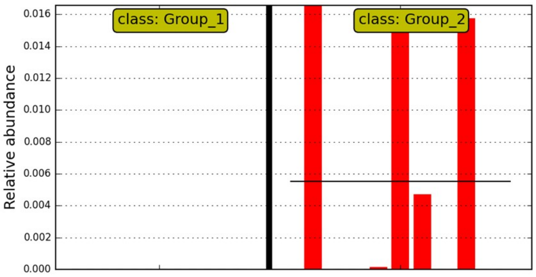

AN Bacteria.Proteobacteria.Epsilonproteobacteria.Campylobacterales.Campylobacteraceae.Campylobacter.Multi_affiliation.Cluster_23

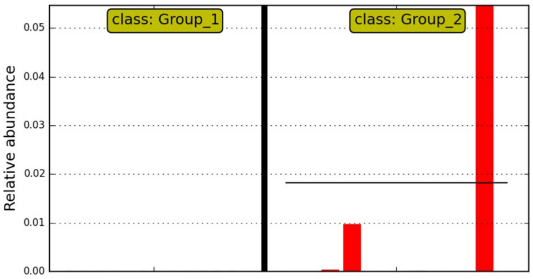

AO Bacteria.Proteobacteria.Gammaproteobacteria

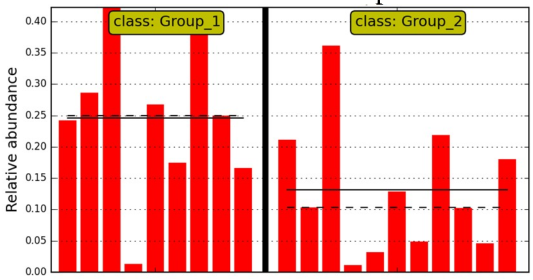

AP Bacteria.Proteobacteria.Gammaproteobacteria.Oceanospirillales.Alcanivoracaceae

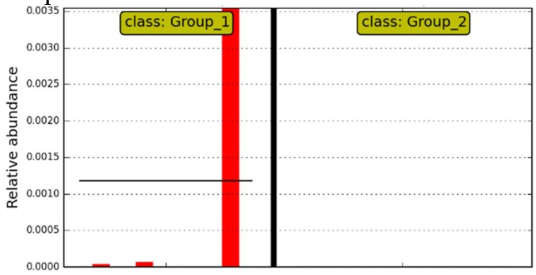

AQ Bacteria.Proteobacteria.Gammaproteobacteria.Oceanospirillales.Alcanivoracaceae.Alcanivorax

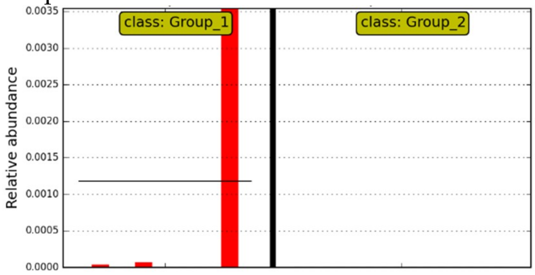

AR Bacteria.Proteobacteria.Gammaproteobacteria.Oceanospirillales.Alcanivoracaceae.Alcanivorax.Unknown

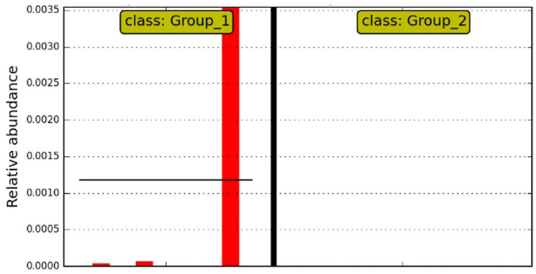

AS Bacteria.Proteobacteria.Gammaproteobacteria.Oceanospirillales.Alcanivoracaceae.Alcanivorax.Unknown.Cluster_198

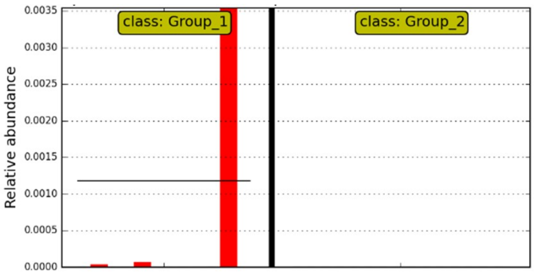

AT Bacteria.Proteobacteria.Gammaproteobacteria.Pseudomonadales

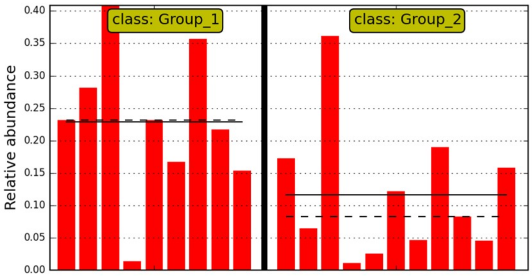

AU Bacteria.Proteobacteria.Gammaproteobacteria.Pseudomonadales.Pseudomonadaceae

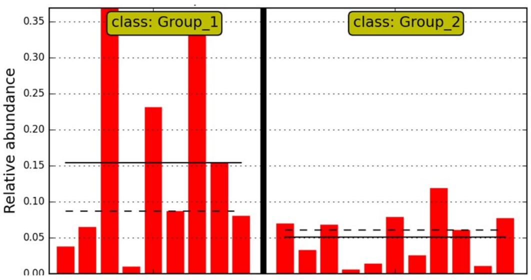

AV Bacteria.Proteobacteria.Gammaproteobacteria.Pseudomonadales.Pseudomonadaceae.Pseudomonas

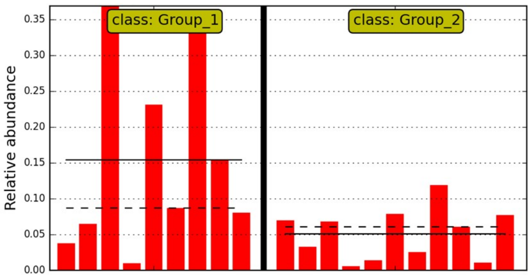

AW Bacteria.Proteobacteria.Gammaproteobacteria.Pseudomonadales.Pseudomonadaceae.Pseudomonas.Multi_affiliation

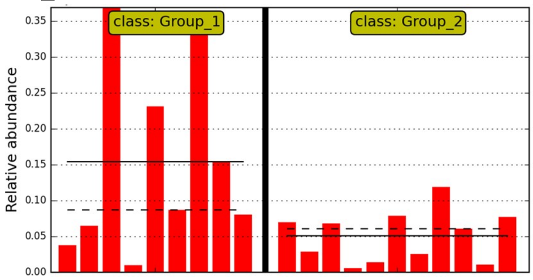

AX Bacteria.Synergistetes

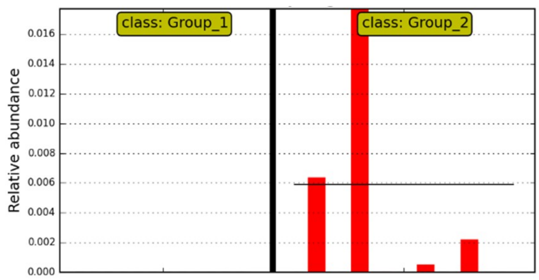

AY Bacteria.Synergistetes.Synergistia

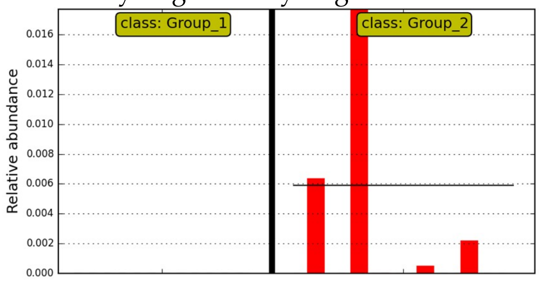

AZ Bacteria.Synergistetes.Synergistia.Synergistales

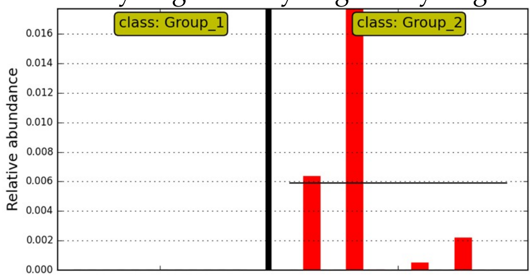

AAA Bacteria.Synergistetes.Synergistia.Synergistales.Synergistaceae

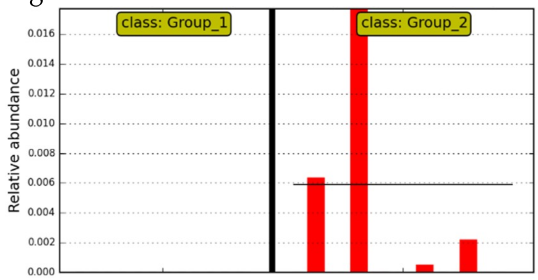

Figure S3: A–AAA Barplots show the abundances of the genera and families between the ESRD-PD and ESRD-nonPD. Each bar represents a sample. Group 1 – End-stage renal disease patients undergoing peritoneal dialysis (ESRD-PD); Group 2 – End-stage renal disease patients with intact peritoneal cavity (ESRD-nonPD).

Figure S4

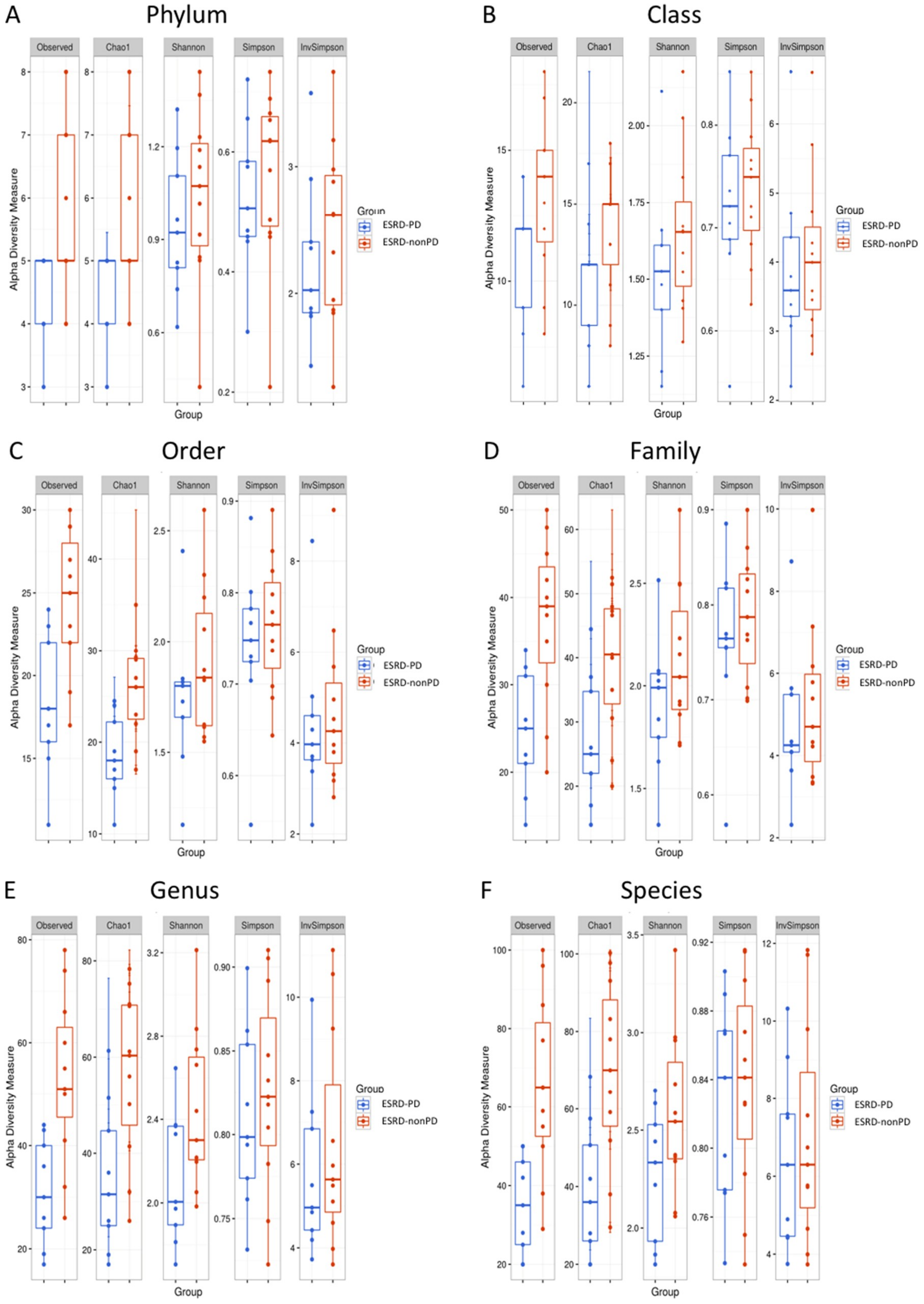
A
Phylum
B
Class
C
Order
D
Family
E
Genus
F
Species
Observed
Chao1
Shannon
Simpson
InvSimpson
Alpha Diversity Measure
Group
Group
ESRD-PD
ESRD-nonPD

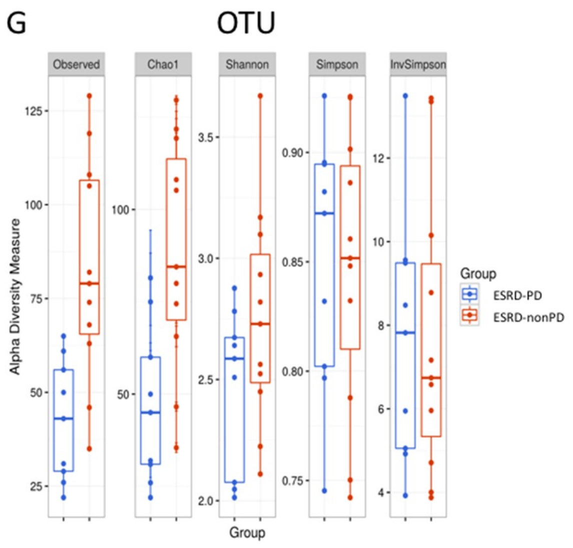

Figure S4: Alpha diversity of the peritoneum microbiome community at phylum (A), class (B), order (C), family (D), genus (E), species (F) and OUT (G) taxonomic levels calculated with Observed, Chao1, Shannon, Simpson, and Inverse Simpson indexes. ESRD-PD – End-stage renal disease patients undergoing peritoneal dialysis; ESRD-nonPD – End-stage renal disease patients with intact peritoneal cavity.

Figure S5

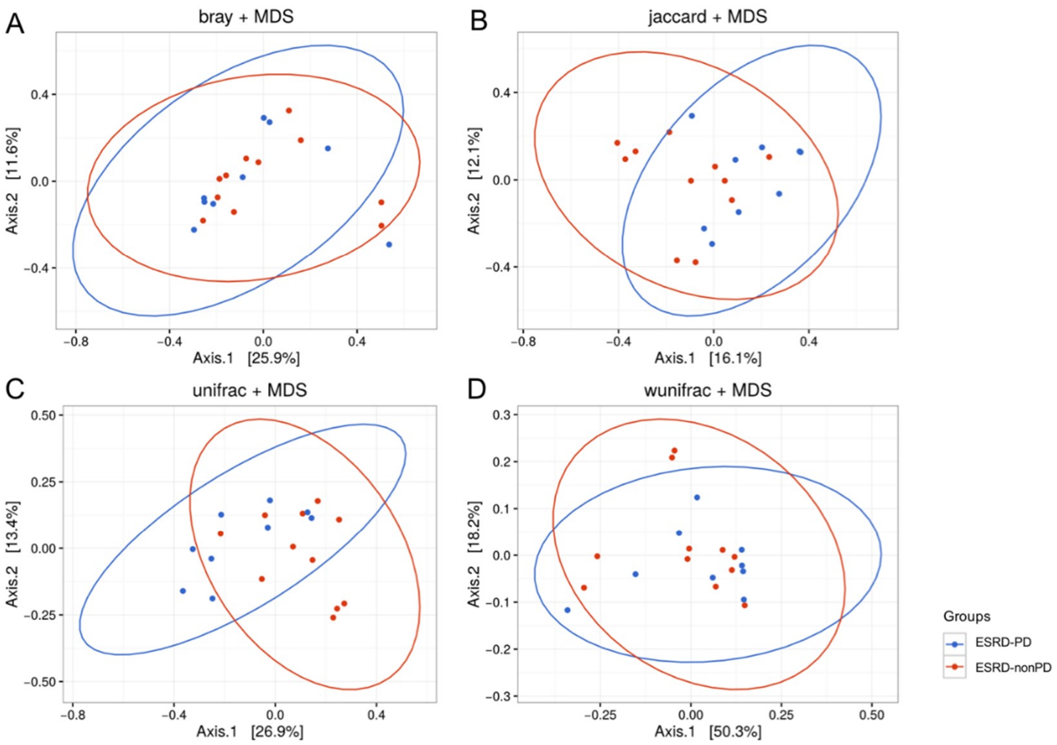

Figure S5: Beta-diversity of the peritoneum microbiome community at OTU level with Bray-Curtis (A), Jaccard (B), unweighted Unifrac (C), and Weighted Unifrac (D) distance derived from 16S rRNA gene -based sequencing data. ESRD-PD – End-stage renal disease patients undergoing peritoneal dialysis; ESRD-nonPD – End-stage renal disease patients with intact peritoneal cavity.

Figure S6

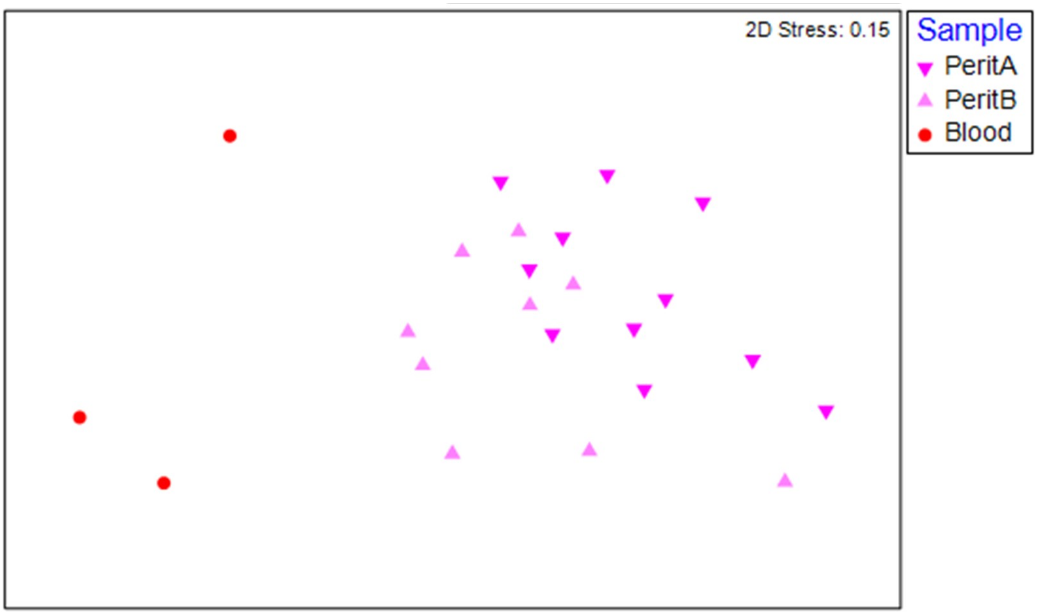

Figure S6: Non-metric multidimensional scaling (NMDS) of blood (red sphere) and peritoneum microbiomes of non-PD (pink triangles) and PD (light pink triangles) patients described in this study. The graph was produced in Primer 7 software using the microbiome information for the genera (total reads per taxa were converted to percentage for each sample), squared-root transformed data and using resemblance matrices (similarity data types using Bray-Curtis similarities and adding dummy value).
